# Structural and Optical Properties of Tungsten Disulfide Nanoscale Films Grown by Sulfurization from W and WO_3_

**DOI:** 10.3390/nano13071276

**Published:** 2023-04-04

**Authors:** Pangihutan Gultom, Jiang-Yan Chiang, Tzu-Tai Huang, Jung-Chuan Lee, Shu-Hsuan Su, Jung-Chung Andrew Huang

**Affiliations:** 1 Department of Physics, National Cheng Kung University, Tainan 701, Taiwan; pangihutangultom36@gmail.com (P.G.); b36355602@gmail.com (J.-Y.C.); asd4561377@gmail.com (T.-T.H.); leejungchuan@gmail.com (J.-C.L.); macg0510@yahoo.com.tw (S.-H.S.); 2 Department of Applied Physics, National University of Kaohsiung, Kaohsiung 811, Taiwan; 3 Taiwan Consortium of Emergent Crystalline Materials, Ministry of Science and Technology, Taipei 10601, Taiwan

**Keywords:** WS_2_, ion beam sputtering technique, W-metal and WO_3_ sulfurization, monolayer crystal structure, optical properties

## Abstract

Tungsten disulfide (WS_2_) was prepared from W metal and WO_3_ by ion beam sputtering and sulfurization in a different number of layers, including monolayer, bilayer, six-layer, and nine-layer. To obtain better crystallinity, the nine-layer of WS_2_ was also prepared from W metal and sulfurized in a furnace at different temperatures (800, 850, 900, and 950 °C). X-ray diffraction revealed that WS_2_ has a 2-H crystal structure and the crystallinity improved with increasing sulfurization temperature, while the crystallinity of WS_2_ sulfurized from WO_3_ (WS_2_-WO_3_) is better than that sulfurized from W-metal (WS_2_-W). Raman spectra show that the full-width at half maximum (FWHM) of WS_2_-WO_3_ is narrower than that of WS_2_-W. We demonstrate that high-quality monocrystalline WS_2_ thin films can be prepared at wafer scale by sulfurization of WO_3_. The photoluminescence of the WS_2_ monolayer is strongly enhanced and centered at 1.98 eV. The transmittance of the WS_2_ monolayer exceeds 80%, and the measured band gap is 1.9 eV, as shown by ultraviolet-visible-infrared spectroscopy.

## 1. Introduction

Tungsten disulfide (WS_2_) is a two-dimensional (2-D) material consisting of a covalently bonded sheet of W atoms filled between two trigonal sheets of S atoms. WS_2_ is mainly composed of three structures, hexagonal (2-H), trigonal (1T), or rhombohedral (3R) phase. Among them, the 2H phase structure is relatively stable and exhibits better optical properties [1]. The interlayers of WS_2_ are bound only by weak van der Waals forces, and the interlayer spacing is ~0.6 nm [2,3].

WS_2_ has been extensively studied due to its unique layer-dependent properties, such as the ability to absorb 5–10% of incident sunlight [4], and its unique band structure. Monolayer WS_2_ exhibits a direct band gap [5] of 1.98 eV [6], while multilayer WS_2_ shows an indirect band gap [7] of about 1.3 eV [8,9]. This phenomenon can be attributed to the lack of Coulomb repulsion between the p_z_ orbitals of the chalcogenide elements in adjacent layers, leading to stabilization of the Γ-state valence band [10]. WS_2_ has been investigated for applications such as solar cells [11], hydrogen evolution reactions (HER) [12], electrocatalysis [13], batteries [14], and transistors [15]. However, the efficiency of most of the devices remains low. Therefore, a better understanding of the formation and physical properties of WS_2_ is essential. Many efforts have been made to improve the application value of WS_2_, such as controlling the formation mechanism, including studying the growth mode of the films to obtain a uniform, large-area [16], and well-oriented crystals. There are three common methods for depositing WS_2_ thin films: the stripping method [17], the chemical vapor deposition (CVD) [18,19], and the direct vulcanization synthesis method [20]. In this study, we seek to obtain high-quality WS_2_ films by comparing tungsten (W) metal and oxide (WO_3_) sulfurization processes. The structural and optical properties of the WS_2_ thin films were investigated in detail for future applications in next-generation optoelectronic devices.

## 2. Experimental

The W and WO_3_ films were prepared on c-axis Al_2_O_3_ (1 cm^2^, provided by Bangjie Material Technology). First, the Al_2_O_3_ substrate was ultrasonicated in acetone for 5 min to remove dirt and then rinsed with methanol. The cleaned c-axis sapphire was then inserted into the ion beam sputtering (IBS, Commonwealth Scientific) chamber for the growth of W or WO_3_ films. Subsequently, the grown W or WO_3_ films were sulfurized to obtain WS_2_ films, as schematically shown in Figure S1a,b of the Supplementary Document. The sputtering of W films was performed at a base pressure of about 5 × 10^−6^ Torr. Ar (99.999% purity) was introduced into the chamber at a flow rate of 5 sccm to initiate sputtering process. On the other hand, the WO_3_ films were grown by flowing Ar and oxygen at flow rates of 5 and 3 sccm, respectively. The sulfurization of the W and WO_3_ films was carried out in a horizontal quartz-tube furnace. The W or WO_3_ films were placed in the center of the quartz-tube furnace while about 3 g of sulfur (99.999% purity) was placed next to the tube lid. The tube was then evacuated to 5 × 10^−2^ Torr. During the sulfurization process, nitrogen gas (99.999%) flowed into the chamber while maintaining a pressure of 0.7 Torr. In this work, the sulfurization process is maintained at 900 °C for thickness-dependent studies. To study the crystallinity behavior, the nine-layer sample of WS_2_-W was sulfurized at different temperatures of 800, 850, 900, and 950 °C. All sulfurization processes were carried out for 20–30 min and cooled naturally to room temperature. The elemental compositions of the WS_2_ films were examined using X-ray photoelectron spectroscopy (XPS, Thermo Fisher Scientific with an Al Kα light source). The binding energies were referenced to the NIST-XPS database. The WS_2_ phase was identified by micro-Raman (Horiba Jobin Yvon Lab RAM HR) equipped with an 1800-cycle grating, an objective lens with 100× magnification, and a laser wavelength of 532 nm. X-ray absorption near-edge spectroscopy (XANES) was performed to identify the local electron structure of WS_2_. The XANES was performed at the 07A1 beamline of the National Synchrotron Radiation Research Center (Hsinchu, Taiwan). The crystal structure of WS_2_ films was examined by X-ray diffraction (XRD, D8 by Bruker AXS Gmbh) equipped with a Cu 2 keV light source. A high-resolution transmission electron microscope (HR-TEM, JEOL JEM-2100F CS STEM) equipped with a dual-beam focus ion beam was conducted to study the microstructure and thickness at the atomic scale. The electrons were accelerated at 12 kV and a magnification of 500 K. The TEM was also equipped with an energy-dispersive X-ray spectrometer (EDS). Further analysis was performed using micro-photoluminescence spectroscopy (PL) to study the band gap of WS_2_ and ultraviolet-visible-infrared spectroscopy to understand the light absorption properties of the samples.

## 3. Results and Discussion

### 3.1. Structural Analysis

**Figure 1:**
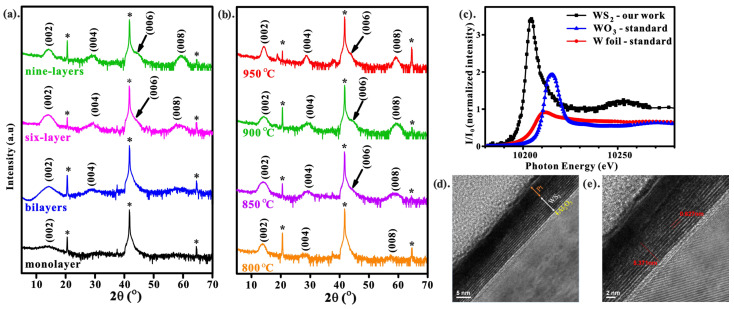
Structure and morphology of WS_2_. The XRD spectra of WS_2_ films for (**a**) different numbers of layers; (**b**) different sulfurization temperatures of nine-layer of WS_2_; (**c**) the morphology of the WS_2_ is presented by TEM image; (**d**) the HR-TEM shows the interlayer distance of WS_2_ stacking layer; (**e**) the XANES shows the local structure of WS_2_.

Figure 1a,b shows the XRD of WS_2_-W at different sputtering thicknesses and sulfurization temperatures. All the XRD peaks marked with an asterisk match the substrate Al_2_O_3_ peaks. The presence of lattice planes (002), (004), (006), and (008) suggests that the structure of WS_2_ is a 2H (hexagonal) phase [5]. Moreover, the interlayer spacing of the WS_2_ was estimated to be ~0.62 nm using the Bragg equation, which agrees with the theory and previous results [21,22].

With increasing film thickness, the intensity of XRD signals (002), (004), (006), and (008) increases, while the FWHM of each peak is reduced, as shown in Figure 1a, indicating that the crystallinity of the films improves with the number of WS_2_ layers. The absence of the (002) peak for the monolayer of WS_2_ is due to the diffraction limit. The tungsten films were sulfurized at different temperatures of 800, 850, 900, and 950 °C to obtain the optimum temperature for formation WS_2_ (nine-layer sample). The corresponding XRD results are shown in Figure 1b, indicating that the crystallinity of WS_2_ improves with increasing sulfurization temperature. Additionally, to study the local structure of WS_2_, the X-ray absorption near-edge structure (XANES) was also performed at W L_3_-edge. Figure 1c exhibits the spectrum of WS_2_ with tungsten and tungsten trioxide as references. The XANES of WS_2_ agrees with the previous result [23]. In addition, it also confirms that the WS_2_ (black path) has a 2H (hexagonal) structure.

TEM investigation has been carried out to observe the atomic stacking of WS_2_ (nine-layer sample). In order to improve the conductivity of the film, about 5 nm of platinum was deposited on the surface of WS_2_. Platinum was also used to protect the sample from oxidation. Figure 1c shows the TEM image of the nine-layer of WS_2_ stacked along the c-axis with the platinum capping layer. The total thickness of the WS_2_ layer was estimated to be ~5.37 nm with an interplanar spacing of 0.62 nm (Figure 1d), which agrees with the XRD result. 

### 3.2. Elemental Composition Analysis

**Figure 2:**
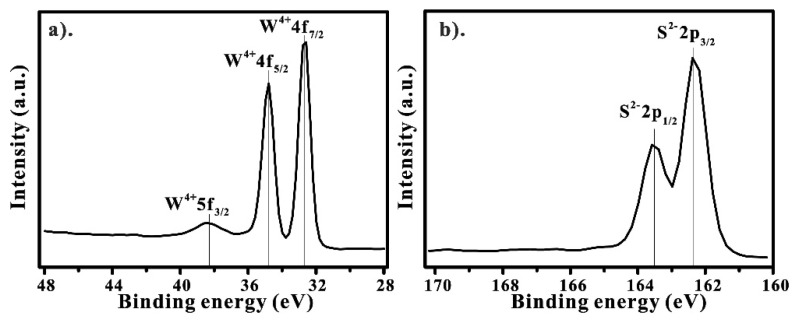
High-resolution XPS of WS_2_ films: (**a**) W 4f and W 5f signals; (**b**) S 2p.

The elemental composition of WS_2_ was qualitatively examined by X-ray photoelectron spectroscopy (XPS). Figure 2 shows high-resolution XPS spectra in W^4+^ and S^2−^ energy regions. Figure 2a exhibits the tungsten signals at 32.6, 34.8, and 38.2 eV, which can be ascribed to W 4f_7/2_, W 4f_5/2_, and W 5f_3/2_, respectively, with a spin-orbit splitting of $\Delta E_{P}\;$(4f_7/2_ − 4f_5/2_) = 2.2 eV. The existence of high-intensity signals at 32.6 and 34.8 eV also suggests the formation of 2H-WS_2_, which concurs with previous reports [24,25]. Figure 2b shows the spectrum of the doublet S 2p at 163.5 eV and 162.3 eV, representing S 2p_1/2_ and S 2p_3/2_, respectively [26,27]. These peaks are attributed to the divalent sulfide ions (S^2−^), which are also associated with the formation of 2H-WS_2_. The elemental composition of WS_2_ was estimated to be 56.0, 28.7, and 15.3% (in atomic percentage) for S 2p, W 4f, and O 1s, in order. The oxygen signal is from the sapphire substrate. Therefore, the W and S ratio is very close to 2:1, confirming that the samples are in good stoichiometry. Additionally, the elemental composition of WS_2_ was also qualitatively examined using EDS (Figure S2).

### 3.3. Optical Properties

#### 3.3.1. Micro-PL Spectroscopy

Micro-PL has been performed to study the optical and electronic properties of WS_2_. It is found that the PL signal comes from the energy gap of the direct transition when the thickness is reduced below two atomic layers. The PL of WS_2_ is strongly suppressed as thickness increases above two atomic layers, in which the indirect band gap is completely formed.

**Figure 3:**
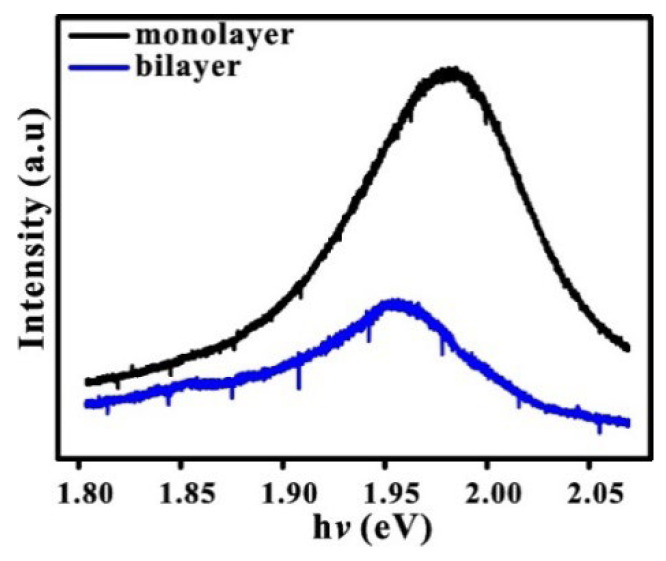
Photoluminescence spectra of WS_2_ monolayer and bilayer.

Figure 3 shows the PL peaks of the monolayer and bilayer of WS_2_. The peaks are centered at 1.98 and 1.95 eV, respectively, in good agreement with previous results (2.1–1.9 eV) [28] and with the DFT-LDA calculations for the monolayer and the bilayer of WS_2_ [29,30]. The PL intensity shows a slight redshift (~3 eV) and a threefold decrease in intensity as the layer increases from the monolayer to the bilayer of WS_2_ [3]. The peak shift is likely due to the proportional relationship between layer number and carrier concentration, which also contributes to Coulomb scattering [31]. This result is in line with the theoretical prediction that the WS_2_ monolayer has a direct band gap at the Γ point [32].

#### 3.3.2. Ultraviolet-Visible-Infrared Spectroscopy

**Figure 4:**
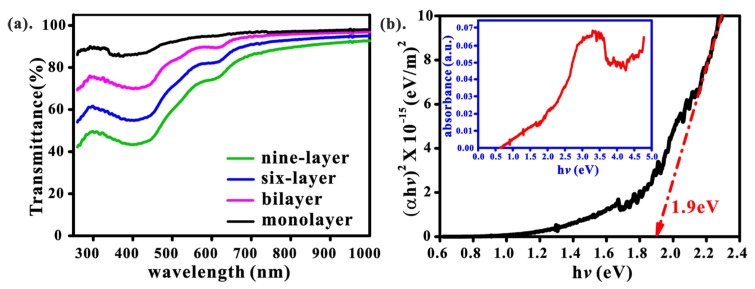
(**a**) Comparison of the transmittance of the WS_2_ thin film with different thicknesses; (**b**) Tauc diagram of monolayer WS_2_ from the absorption. Inset: the absorption measurement can be used to determine the band gap of WS_2_*_._*

The optical properties of WS_2_ were further investigated using an ultraviolet-visible-infrared (UV/visible/NIR) spectrophotometer, as shown in Figure 4. It was observed that the transmittance of WS_2_ increased from 40% (nine-layer) to over 80% (monolayer) in the UV-visible region (Figure 4a). The band gap of the WS_2_ monolayer is about 1.9 eV, as estimated from the Tauc plot of the absorption spectrum (Figure 4b).

### 3.4. Comparison of WS_2_-W and WS_2_-WO_3_

#### 3.4.1. XRD of WS_2_-W and WS_2_-WO_3_

**Figure 5:**
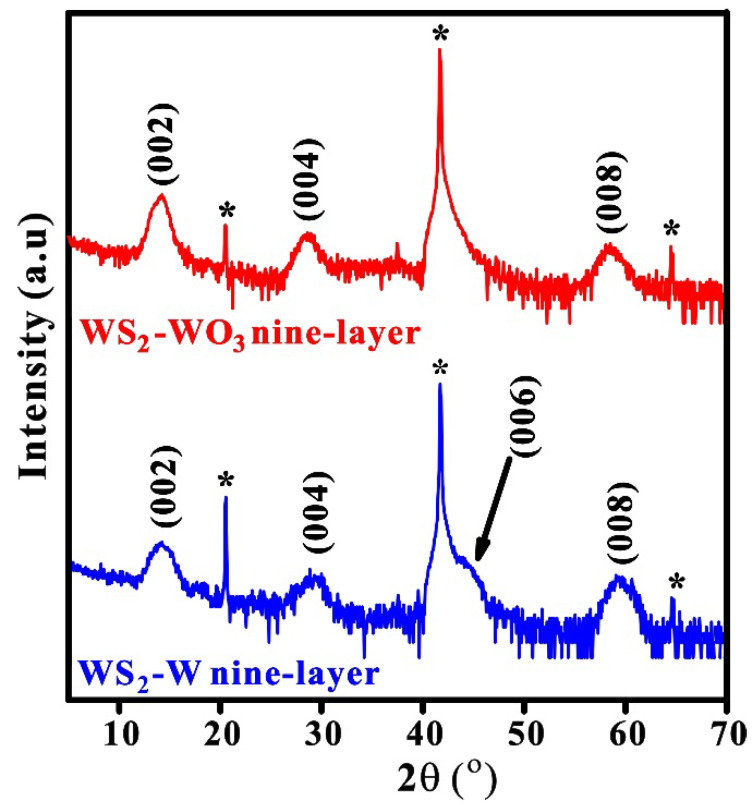
XRD of nine-layer WS_2_ sulfurized from W and WO_3_ at 900 °C It shows narrower FWHMs of 002, 004, and 008 signals from WS_2_-WO_3_ (2.1 ± 0.1; 3.4 ± 0.2; 2.6 ± 0.2, respectively) then from WS_2_-W (2.2 ± 0.1; 4.1 ± 0.2; 3.1 ± 0.2, respectively).

In order to obtain high-quality WS_2_ films, we compared the sulfurization of W and WO_3_ films, termed as WS_2_-W and WS_2_-WO_3_, respectively. The samples were sulfurized side by side at 900 °C. The XRD results are shown in Figure 5. It was revealed that the diffraction peaks of WS_2_-WO_3_ are sharper and narrower than those of WS_2_-W with similar thickness, suggesting that the crystallinity of WS_2_-WO_3_ is better than that of WS_2_-W.

#### 3.4.2. Raman Spectra of WS_2_-W and WS_2_-WO_3_

**Figure 6:**
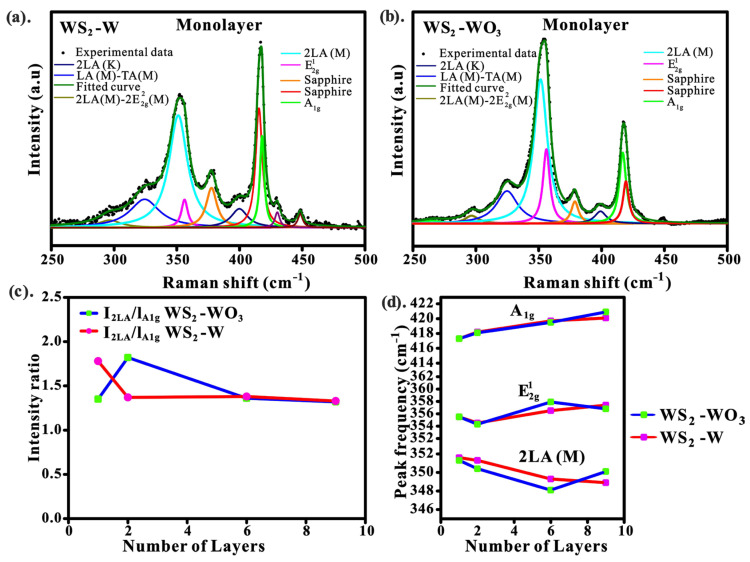
(**a**,**b**). Deconvolution of the Raman spectra of WS_2_-W and WS_2_-WO_3_, respectively, using Lorentz fitting method; (**c**) the intensity ratio of $I_{2LA}/I_{A_{1g}}$ as a function of the number layers of WS_2_; (**d**) the peak positions of *A_1g_*, $E_{2g}^{1}$, and 2LA with respect to the number layer of WS_2_. Additionally, the deconvolution of the Raman spectra for bilayer, six-layer, nine-layer can be seen in the Supplementary Documents.

Raman spectroscopy is widely used for elemental analysis, layer stacking order, and doping effect of transition metal dichalcogenides [33]. Figure 6 shows the Raman spectra of WS_2_-W and WS_2_-WO_3_ with the different number of stacked layers.

Figure 6a,b shows two main Raman peaks centered at ~354 and ~420 cm^−1^, respectively. These Raman peaks agree with previous work [21,34,35]. It was observed that the *A_1g_* peaks overlap with the Raman peaks of sapphire (380 and 417cm^−1^) [36] when the Raman peaks were deconvoluted using a multi-peak Lorentzian fitting method [37]. Deconvolution of Raman peaks also revealed three strong peaks, which are $E_{2g}^{1}$ (in-plane displacement of W and S), *A_1g_* (out-plane displacement of S-S atoms), and 2LA (M) (second order Raman peak), which are centered at ~356, ~418, and ~351 cm^−1^_,_ respectively. Furthermore, the approximate distance between *A_1g_* and $E_{2g}^{1}$ of WS_2_-W is ~61.8, 63.8, 61.6, and 64.1 cm^−1^ for monolayer, bilayer, six-layer, and nine-layer samples, respectively, which is not much different from the distance for WS_2_-WO_3_ of about 61.8, 63.7, 63.2, 62.7 in the same order. Figure 6c shows the $I_{2LA}/I_{A_{1g}}$ intensity ratio of WS_2_-W reduces from the monolayer (1.8) to the nine-layer (1.3). However, WS_2_-WO_3_ shows a slightly different trend, where the intensity ratio increases from the monolayer (1.3) to the bilayer (1.8), and then decreases to the nine-layer (1.3). The results suggest that the second-order Raman peak intensity ($I_{2LA}$) is almost twice that of the first-order $\;I_{A_{1g}}$. This is consistent with the previous results [38,39]. Additionally, for both WS_2_-W and WS_2_-WO_3_ samples, as the film thickness decreases, the *A_1g_* signals show a slight red shift of about ~2.3 cm^−1^ (Figure 6d). The redshift of *A_1g_* is likely associated with reducing the atomic restoring forces. When the number of WS_2_ layers is decreased, the long-range Coulomb interaction between effective charges and dielectric screening is enhanced, leading to increased restoring force between the S-S atoms [3].

Due to the dielectric screening effect, the Raman signal is sensitive to the interactions between atoms in the interlayer and long-range Coulomb interactions [40]. The Raman wavevector and the intensity of WS_2_ are strongly correlated with the layer thickness at the nanoscale level. In this work, we also used Raman spectra to calibrate the thickness of WS_2_ as a monolayer, bilayer, six-layer, and nine-layer [31,39]. The Raman spectra confirmed that WS_2_ was well-formed as WS_2_-W and WS_2_-WO_3_. It is clear that the peak intensity and FWHM of $E_{2g}^{1}$ for WS_2_-WO_3_ are narrower than that of WS_2_-W (Table S1 in the Supplementary Materials). The average estimation of FWHM of WS_2_-WO_3_ is ~7.2; however, the WS_2_-W is ~10.1, suggesting WS_2_-WO_3_ has better crystallinity than WS_2_-W. This is consistent with the XRD results (Figure 5). This phenomenon can be attributed to the fact that sulfur can replace oxygen more effectively at a relatively lower temperature [41], while WS_2_ formed from tungsten metal requires a higher temperature to facilitate metal–sulfur bonding [42]. As a result, the defect density in WS_2_-WO_3_ is likely to be lower than in WS_2_-W.

## 4. Conclusions

We report a study of nanoscale WS_2_ films with various number of layers, prepared by sulfurization of W and WO_3_ at different temperatures (800 to 950 °C). The WS_2_ films were inferred to be the 2H phase and c-axis oriented. The XRD shows that the crystal quality of the WS_2_ films improved with increasing sulfurization temperature. The photoluminescence of the monolayer of WS_2_ is strongly enhanced and centered at 1.98 eV. The transmittance of the monolayer WS_2_ exceeds 80% and the band gap is 1.9 eV, revealed by ultraviolet-visible-infrared spectroscopy. The Raman analysis shows that the FWHM of WS_2_-WO_3_ is narrower than that of WS_2_-W, indicating the structure of WS_2_-WO_3_ is superior to that of WS_2_-W, which is in good agreement with the X-ray diffraction result. We conclude that a large-area, high-quality WS_2_ film can be prepared by sulfurizing WO_3_. The results are promising for applications in next-generation optoelectronic devices.

## Data Availability

All data included in this study are available upon request by contact with the corresponding author.

## Supplementary Materials

The following supporting information can be downloaded at: https://www.mdpi.com/article/10.3390/nano13071276/s1, Figure S1: Schematic diagram: (a). W-metal and WO_3_ prepared on sapphire substrate by ion beam sputtering technique; (b). W-metal or WO_3_ sulfurization process using a thermocouple-equipped furnace, the process was carried out inside a horizontal quartz tube with a diameter of 50 mm and length of 100 cm; Figure S2: shows the EDS depth analysis of the WS_2_ film that was carried out with Line scan mode. The small picture in the upper left corner is the relationship between the scanning position and the ratio of each element; Figure S3: the deconvolution of Raman spectra of bilayer, six-layer, and nine-layer samples using a multi-peak Lorentz fitting to separate the A1g peak from the substrate and 2LA from E_2 g^1^. The (a–c) shows the WS2 sulfurized from tungsten metal and (d–f) shows the WS2 sulfurized from tungsten trioxide; Table S1: Summary of the intensity ratio of $I_{2LA}/I_{A_{1g}}$ and FWHM as a function of layer numbers.
