# Supplementary material for: Structural and Optical Properties of Tungsten Disulfide Nanoscale Films Grown by Sulfurization from W and WO3
Source: Nanomaterials (Basel). 2023 Apr 4;13(7):1276. doi: 10.3390/nano13071276 (PMC10096497; doi:10.3390/nano13071276)
Supplement: Supplementary file 1 [file nanomaterials-13-01276-s001.zip › nanomaterials-2263251-supplementary.pdf]

nanomaterials

Supplementary Materials

Structural and Optical Properties of Tungsten Disulfide Nanoscale Films Grown by Sulfurization from W and WO_3

Pangihutan Gultom [1], Jiang-Yan Chiang [1], Tzu-Tai Huang [1], Jung-Chuan Lee [1], Shu Hsuan Su [1] and Jung-Chung Andrew Huang [1,2,3*]

1 Department of Physics, National Cheng Kung University, Taiwan 701, Taiwan; pangihutan-gultom36@gmail.com (P.G.); b36355602@gmail.com (J.-Y.C.); asd4561377@gmail.com (T.-T.H.); leejungchuan@gmail.com (J.-C.L.); macg0510@yahoo.com.tw (S.-H.S.)
2 Department of Applied Physics, National University of Kaohsiung, Kaohsiung 811, Taiwan
3 Taiwan Consortium of Emergent Crystalline Materials, Ministry of Science and Technology, Taipei 10601, Taiwan
* Correspondence: author: jcahuang@mail.ncku.edu.tw; Tel.: +886-6-2757575.

Preparation of WS_2

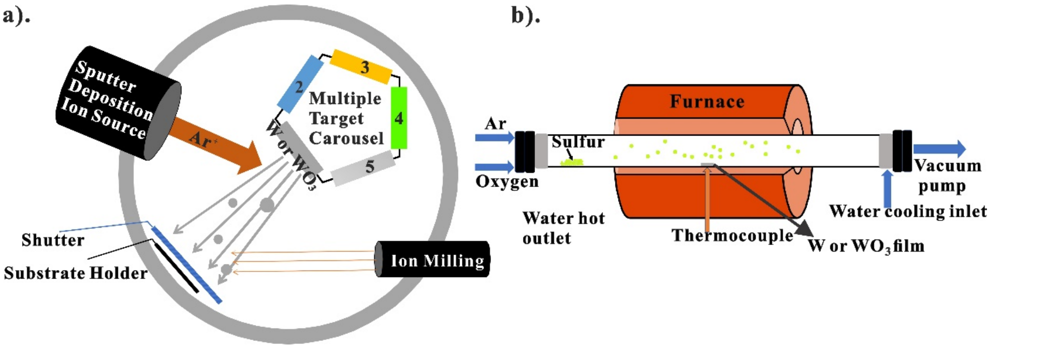

Figure S1. Schematic diagram: **a**). W-metal and WO_3 prepared on sapphire substrate by ion beam sputtering technique; **b**). W-metal or WO_3 sulfurization process using a thermocouple-equipped furnace, the process was carried out inside a horizontal quartz tube with a diameter of 50 mm and length of 100 cm.

Energy dispersive X-ray spectroscopy (EDS)

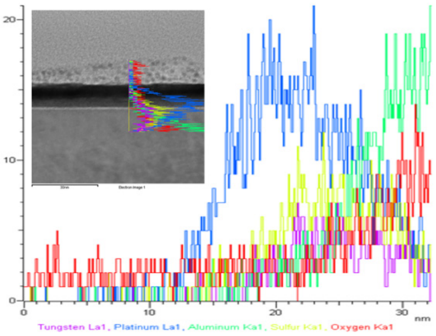

Figure S2. shows the EDS depth analysis of the WS_2 film that was carried out with Line scan mode. The small picture in the upper left corner is the relationship between the scanning position and the ratio of each element.

Figure S2 EDS elemental analysis of the WS_2 films, there are five main elements: tungsten, platinum, aluminum, sulfur, and oxygen which correspond to the purple, blue, green, yellow, and red paths, respectively.

Raman spectra and deconvolution

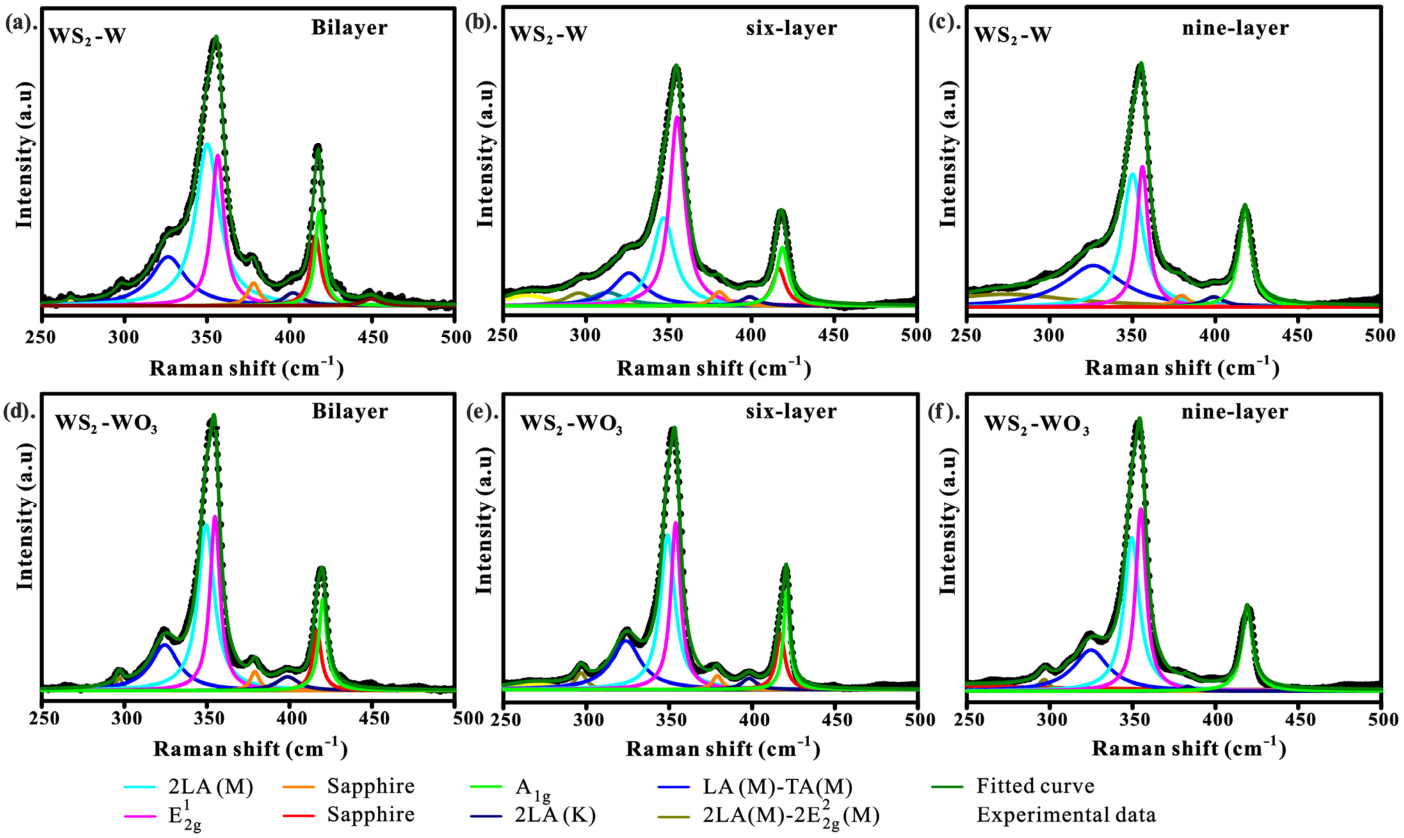

Figure S3. the deconvolution of Raman spectra of bilayer, six-layer, and nine-layer samples using a multi-peak Lorentz fitting to separate the A_{1g} peak from the substrate and 2LA from E_{2g}^{1}. The (**a**–**c**) shows the WS_2 sulfurized from tungsten metal and (**d**–**f**) shows the WS_2 sulfurized from tungsten trioxide.

Table S1. Summary of the intensity ratio of $I_{2LA}/I_{A_{1g}}$ and FWHM as a function of layer numbers.

Number of layers	$I_{E_{2g}^{1}}/I_{A_{1g}}$		FWHM of E_{2g}^{1}	
	WS_2-W	WS_2-WO_3	WS_2-W	WS_2-WO_3
Monolayer	1.4	1.8	9.1 ± 0.5	8.6 ± 2.16
Bilayer	1.8	1.5	8.6 ± 0.4	7.1 ± 0.4
Six-layer	1.4	1.4	10.9 ± 0.4	6.6 ± 0. 4
Nine-layer	1.3	1.3	11.6 ± 0.8	7.7 ± 0.3
